# Best urological practices on testing and management of infertile men with abnormal sperm DNA fragmentation levels: the SFRAG guidelines

**DOI:** 10.1590/S1677-5538.IBJU.2020.1004

**Published:** 2020-12-20

**Authors:** Sandro C. Esteves, Armand Zini, Robert Matthew Coward

**Affiliations:** 1 Centro de Referência para Reprodução Masculina Clínica de Andrologia e Reprodução Humana Campinas Brasil ANDROFERT, Clínica de Andrologia e Reprodução Humana, Centro de Referência para Reprodução Masculina, Campinas, SP, Brasil.; 2 Universidade Estadual de Campinas Departamento de Cirurgia (Disciplina de Urologia) CampinasSP Brasil Departamento de Cirurgia (Disciplina de Urologia), Universidade Estadual de Campinas - UNICAMP, Campinas, SP, Brasil.; 3 Aarhus University Faculty of Health Aarhus Denmark Faculty of Health, Aarhus University, Aarhus, Denmark.; 4 McGill University St. Mary’s Hospital Urology Division MontrealQuébec Canada Department of Surgery, Urology Division, St. Mary’s Hospital, McGill University, Montreal, Québec, Canada.; 5 University of North Carolina Department of Urology RaleighNorth Carolina USA Department of Urology, University of North Carolina, Chapel Hill, North Carolina, UNC Fertility, Raleigh, North Carolina, USA.

## INTRODUCTION

The prevention and management of male infertility is an integral component of sexual and reproductive health services. Male factors, alone or combined with female factors, explain up to 50% of infertility cases, and when present, an evaluation by a urologist experienced in diagnosing and treating male factor infertility is highly recommended. In Brazil, like the United States and Canada (1), most patients are referred to urologists by (reproductive) gynecologists based on an abnormal semen analysis result. The work-up involves a detailed medical history and physical examination and, when indicated, hormone, genetic, and imaging tests, all of which are used to guide clinical management (2).

The semen analysis is one of the earliest tests in the infertility work-up. The standard assessment of semen characteristics includes ejaculate volume, sperm count, sperm motility, and sperm morphology. Although informative, they provide limited discriminatory information about the male fertility potential, unless at extremely low levels (3). Recently, increased attention has been given to the evaluation of sperm DNA, whose integrity is indispensable for post-fertilization events and the birth of healthy offspring (4). Infertile men often have abnormal levels of sperm DNA fragmentation (SDF), which is a marker of damaged chromatin (5).

Measurement of SDF in the ejaculated semen is used to obtain information about sperm DNA quality at the molecular level. Sperm DNA breaks can be detected using probes or dyes under fluorescence or optical microscopy or flow cytometry examination. Several interventions have been proposed to mitigate the potential deleterious effect of SDF on reproduction (6, 7). Despite robust evidence relating SDF with infertility, clear guidance on how testing should be performed and to whom it should be offered has been lacking. Moreover, the general belief that high SDF is untreatable has hampered testing in routine clinical practice.

### The sperm DNA fragmentation study group (SFRAG) guidelines

An evidence-based guideline for the investigation and treatment of SDF was published in late 2020 on behalf of the Sperm DNA Fragmentation Study Group (SFRAG) (8). This consensus guideline provides a comprehensive evidence summary about the role of SDF on infertility and offers best practice advice on testing and care of couples confronted with elevated SDF. Furthermore, the guideline provides an overview of the treatments currently available for mitigating elevated SDF, and which ones may be recommended. Recommendations are also formulated on what test should be used and how testing should be conducted to select patients for possible therapeutic interventions.

**Figure 1:**
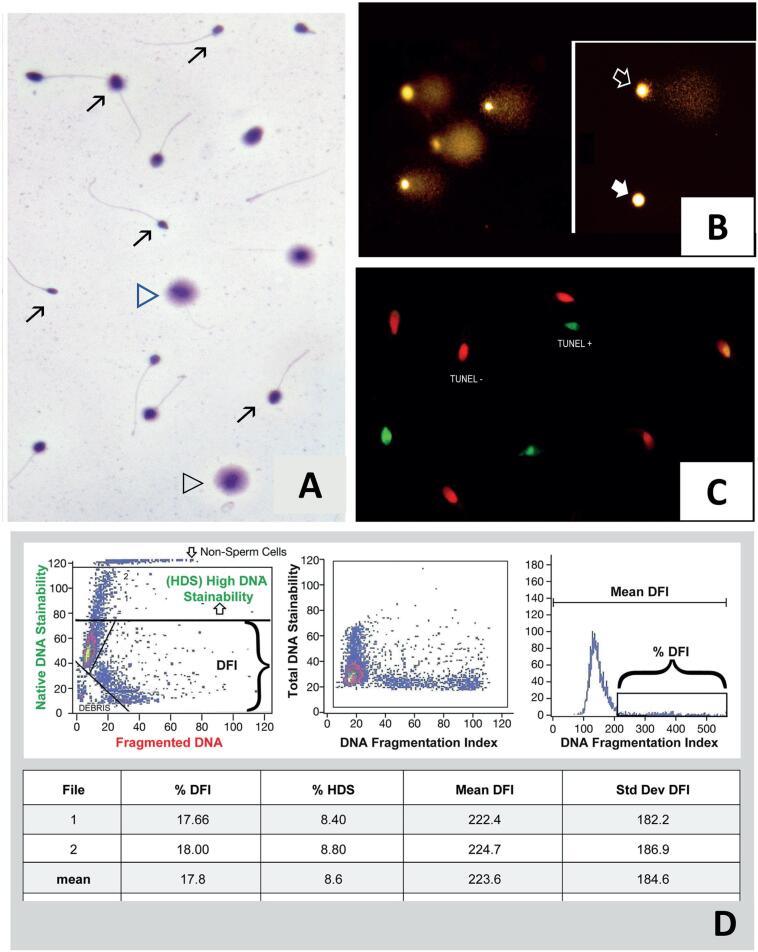
Sperm DNA fragmentation tests. **A) Sperm Chromatin Dispersion test (SCD): Sperm sample of a patient with varicocele presenting with elevated SDF. Open arrowheads indicate sperm with halos of dispersed chromatin representing a normal DNA molecule with no fragmented DNA. Black arrowheads indicate sperm with small or absent halos of dispersed chromatin, representing sperm with fragmented DNA. Arrows in indicate sperm with no halos at all, fragmented-degraded DNA. B) Alkaline Comet assay under fluorescence microscopy: Sperm sample of a patient exhibiting elevated sperm DNA fragmentation (SDF). Several comets are shown, which represent sperm with DNA fragmentation. The longer and brighter the ‘Comet’ tail, the more fragmentation is present. Open arrow: spermatozoon with DNA fragmentation. White arrow: spermatozoon with a hardly visible ‘Comet’ tail, representing a cell with minimal DNA fragmentation. As the Comet test measures the amount of damage in each cell, it is rare to find a perfect spermatozoon with 0% damage, even from fertile donors. C) TUNEL Assay: Visualization of sperm DNA damage using terminal deoxynucleotidyl transferase dUTP nick end labeling (TUNEL). Digoxigenin-dUTP was incorporated into DNA breaks using a terminal transferase that was detected using anti-digoxigenin-FITC (green color). TUNEL+ represents sperm presenting DNA damage. Slides were counterstained with propidium iodide (red color). TUNEL- represents sperm free of DNA breaks. D) Sperm Chromatin Structure Assay (SCSA): Test data (SCSA Diagnostics, Brookings, USA). Left panel (top box): raw data from a flow cytometer showing each of 5.000 sperm as a single dot on a scattergram. Y-axis = green fluorescence with 1.024 gradations (channels) of DNA stainability (intact double-stranded DNA). X-axis = red fluorescence with 1.024 gradations of red fluorescence (single-strand DNA). Axes shown are 1.24/10. The line at Y = 75 marks the upper boundary of DNA staining of normal sperm chromatin; above that line are sperm (dots) with partially uncondensed chromatin allowing more DNA stainability. The bottom left corner shows gating out of seminal debris. Middle panel: Raw data from the left panel are converted by SCSAsoft software (or equivalent) to red/red+green fluorescence. This transforms the angled sperm display in the left panel to a vertical pattern that is often critical for accurately delineating the percentage of sperm with fragmented DNA**. Y-axis = total DNA stainability vs. X-axis = red/red+green fluorescence (DFI). Right panel: Frequency histogram of data from middle panel showing computer gating into %DFI and Mean DFI. Bottom box: SCSAsoft software calculations of the mean of two independent measures of mean and standard deviation (std dev) of median DFI, %DFI, and %HDS (high DNA stainability). Modified from: Esteves SC, Zini A, Coward RM, Evenson DP, Gosálvez J, Lewis SEM, Sharma R, Humaidan P. Sperm DNA fragmentation testing: Summary evidence and clinical practice recommendations. Andrologia. 2020 Oct 27:e13874. Epub ahead of print. This is an open-access article distributed under the Creative Commons Attribution License. The license permits unrestricted use, distribution, reproduction in any medium, remixing, transformation, and building upon the material for any purpose provided the original work is properly cited.

The guideline was developed in three main sections. In the first part, it outlines the SDF pathophysiology and explains each SDF test. This section provides thirteen recommendations on how testing should be carried out and results analyzed (Table-1). Also, a new nomenclature is proposed to classify the sperm chromatin damage tests into two groups, that is, one for the tests that measure SDF (TUNEL, SCSA, SCS, and Comet; Figure-1), and another related to tests that assess chromatin compaction (e.g., chromomycin A3, acridine orange staining, toluidine blue staining, and aniline blue staining).

**Table 1:** Recommendations on technical aspects of Sperm DNA Fragmentation testing, clinical thresholds, and interpretation of results.

Recommendation	GDG strength rating§	OCEBM* recommendation grade based on levels of evidence
The most reliable tests for assessing SDF are SCSA, alkaline Comet, SCD, and TUNEL.	Conditional	Grade B
Any of the four SDF tests (SCSA, alkaline Comet, SCD, and TUNEL) may provide valid information concerning the probability of reproductive success for couples embarking on IUI, IVF, and ICSI.	Conditional	Grade B
A standardized protocol with strict quality control is essential for a reliable SDF testing result. Tests should be validated by the laboratory, with thresholds established based on the evaluation of fertile and infertile populations.	Strong	Grade A-B
A neat semen sample should be used for SDF testing, collected after ejaculatory abstinence of 2-5 days.	Strong	Grade B
Patients should be asked not to have prolonged abstinence periods before the ejaculation that precedes the one used for semen collection and testing.	Conditional	Grade D
A fixed ejaculatory abstinence length should be used for SDF testing when monitoring the effects of medical and surgical interventions aimed at decreasing SDF levels.	Conditional	Grade B
Fresh or frozen-thawed specimens can be used for testing, but the analysis should start as quickly as possible after liquefaction (e.g., 30-60 minutes) or thawing.	Strong	Grade C-D
If a frozen specimen is to be used for SDF testing, freezing should be immediately done after liquefaction is achieved.	Strong	Grade C-D
Overall, thresholds of ~20% (SCSA, TUNEL, and SCD), and 26% (alkaline Comet), best discriminate fertile from infertile men.	Conditional	Grade B
Overall, thresholds exceeding 20–30% (SCSA, alkaline Comet, and SCD) indicate a statistical probability of increased time to achieve natural pregnancy, increased miscarriage risk (after both natural and assisted conception), and low odds of reproductive success by IUI, IVF, and ICSI.	Conditional	Grade B
SDF results –in combination with the current tools for infertility diagnosis– provide useful information concerning the probability of reproductive success.	Conditional	Grade B
SDF tests cannot perfectly discriminate fertile from infertile men or couples that will have a successful IUI, IVF, or ICSI cycle from those that will not.	Strong	Grade B
The usefulness of any test for one partner is also dependent on the fertility of the other partner. Before testing, clinicians should have some understanding of the characteristics of SDF assays (e.g., sensitivity and specificity, positive and negative predictive value).	Strong	Grade B

SDF: sperm DNA fragmentation; ICSI: intracytoplasmic sperm injection; IUI: intrauterine insemination; IVF: in vitro fertilization; SCSA = sperm chromatin structure assay; SCD = sperm chromatin dispersion; TUNEL = Terminal deoxynucleotidyl transferase-mediated dUTP-biotin nick end labeling.

§ Guideline development group (GDG) expert judgment; Strong recommendations imply that most individuals in that situation should receive the testing or intervention. Conditional recommendations imply that different choices might be appropriate for individual patients and that clinicians should help each patient reach a decision consistent with a patient-centered approach.

* Oxford Centre for Evidence-Based Medicine Levels of Evidence (OCEBM Levels of Evidence Working Group)

Grades of recommendations according to quality of evidence:

Grade A: consistent level 1 studies; Grade B: consistent level 2 or 3 studies or extrapolations from level 1 studies; Grade C: level 4 studies or extrapolations from level 2 or 3 studies; Grade D: level 5 or troubling inconsistent or inconclusive studies of any level.

Level 1 studies: systematic reviews with homogeneity of randomized controlled trials (RCTs) or level 1 diagnostic studies (1a); individual RCT with narrow confidence interval or validating cohort studies with good reference standards (2b).

Level 2 studies: systematic reviews with homogeneity of cohort studies or diagnostic studies (2a); individual cohort study or low quality RCT (2b), exploratory cohort study with good reference standards (2b).

Level 3: systematic reviews of case-control studies or moderate quality diagnostic studies (3a), individual case-control studies or non-consecutive diagnostic studies (3b).

Level 4: case-series or poor cohort/case-control studies or case-control diagnostic study.

Level 5: Expert opinion

http://www.cebm.net/oxford-centre-evidence-based-medicine-levels-evidence-march-2009/). Accessed June 7th, 2020.

**Reprinted from:** Esteves SC, Zini A, Coward RM, Evenson DP, Gosálvez J, Lewis SEM, Sharma R, Humaidan P. Sperm DNA fragmentation testing: Summary evidence and clinical practice recommendations. Andrologia. 2020 Oct 27:e13874. Epub ahead of print. This is an open access article distributed under the terms of the Creative Commons Attribution License. The license permits unrestricted use, distribution, reproduction in any medium, provided the original work is properly cited.

The second part details seven clinical situations that may benefit from SDF testing, including i. Varicocele, ii. Unexplained/idiopathic infertility, iii. Recurrent pregnancy loss, iv. Intrauterine insemination, v. In vitro fertilization/intracytoplasmic sperm injection, vi. Infertility risk factors, and vii. Sperm cryopreservation. The guideline provides specific recommendations for each condition-twenty-eight in total (Table-2)- and best practices for treatment. Lastly, the third part lists the main gaps in knowledge and provides recommendations for future research.

**Table 2:** Recommendations on indications for Sperm DNA Fragmentation testing.

Recommendation	GDG strength rating§	OCEBM* recommendation grade based on levels of evidence
Varicocele		
Men with varicocele seeking fertility should be informed that varicocele may cause SDF and that repairing a clinical varicocele may alleviate SDF, potentially increasing the likelihood of reproductive success.	Strong	Grade B-C
SDF testing may help identify patients with a profile that would not fit the standard indication of varicocele repair (e.g., clinical varicocele of any grade and normal/borderline routine semen analysis) but that can benefit from varicocele repair.	Conditional	Grade C
SDF testing may be used to monitor treatment outcomes.	Conditional	Grade C
SDF testing in subfertile men with subclinical varicocele is currently not recommended.	Strong	Grade C
**Unexplained Infertility, Idiopathic Male Infertility, and Recurrent Pregnancy Loss**		
Couples with unexplained infertility, idiopathic infertility, and RPL should be informed that abnormal SDF levels may adversely impact their chances of achieving a live birth.	Strong	Grade B
SDF testing in couples with unexplained infertility, idiopathic infertility, and RPL can be considered for explanatory purposes.	Strong	Grade B-C
An abnormal SDF test result should prompt a complete male evaluation by a reproductive urologist to help identify and possibly treat conditions associated with poor sperm DNA quality.	Strong	Grade D
ICSI may be considered if no correctable male factor is identified, or if abnormal SDF levels persist after treatment, particularly among couples with a limited reproductive time window.	Conditional	Grade B
**Intrauterine Insemination**		
Infertile couples eligible for IUI treatment should be informed that abnormal SDF levels may adversely impact their chances of achieving a live birth.	Strong	Grade B
SDF testing may be considered before initiating IUI or after IUI failure.	Conditional	Grade B-C
An abnormal SDF test result should prompt a complete male evaluation by a reproductive urologist to help identify and possibly treat conditions associated with poor sperm DNA quality.	Strong	Grade D
Early ICSI may be considered in IUI eligible couples, or after failed IUI, if the male partner has high SDF levels, provided other measures to decrease SDF have been exhausted.	Conditional	Grade C
**In Vitro Fertilization/Intracytoplasmic Sperm Injection**		
Infertile couples eligible for conventional IVF treatment should be informed that abnormal SDF levels may adversely impact their chances of achieving a live birth.	Strong	Grade B
Infertile couples eligible for ICSI treatment should be informed that abnormal SDF levels may adversely impact their chances of achieving a live birth.	Conditional	Grade B
SDF testing may be considered before initiating IVF/ICSI or after unexplained failed IVF/ICSI.	Conditional	Grade B-C
An abnormal SDF test result should prompt a complete male evaluation by a reproductive urologist to help identify and possibly treat conditions associated with poor sperm DNA quality.	Strong	Grade D
ICSI rather than conventional IVF should be used to overcome infertility related to SDF.	Strong	Grade B
Among couples with ICSI failure and elevated SDF, testicular rather than ejaculated sperm may be considered for sperm injection in subsequent treatment cycles.	Conditional	Grade B
The use of testicular sperm in preference over ejaculated sperm for ICSI, when both are available, may be particularly relevant for couples with no apparent reasons for a failed ICSI (e.g., no relevant female factors). This advice implies that a reproductive urologist has evaluated the male partner and all possible corrective measures taken to improve overall reproductive health and sperm chromatin integrity.	Conditional	Grade D
**Fertility Counseling for Individuals with Infertility Risk Factors**		
SDF testing may be considered to provide laboratory evidence of defective sperm chromatin to couples who seek fertility counseling and family planning, particularly when the male partner has an infertility risk factor.	Conditional	Grade C
Men with infertility risk factors (e.g., tobacco smoking, obesity, metabolic syndrome, exposure to environmental or occupational toxicants, use of licit or illicit drugs with gonadotoxic effects, and advanced paternal age) should be informed that these factors may cause SDF and that lifestyle changes may alleviate SDF, potentially increasing the likelihood of reproductive success.	Conditional	Grade C
An abnormal SDF test result should prompt a complete male evaluation by a reproductive urologist to help identify and possibly treat conditions associated with poor sperm DNA quality.	Strong	Grade D
An abnormal SDF test result may be used for counseling, reinforcing the importance of lifestyle changes and avoiding exposure to toxins.	Conditional	Grade C
Early ICSI may be considered for individuals with persistently high SDF levels despite corrective interventions, mainly when the reproductive window is limited.	Conditional	Grade D
The information provided by SDF testing may guide the choice of assisted conception modality, IUI, IVF, or ICSI, in infertile couples with a male partner of advanced age.	Conditional	Grade D
SDF testing may be used to monitor the effects of lifestyle interventions.	Conditional	Grade D
**Sperm Cryopreservation**		
SDF testing can be considered before sperm cryopreservation to provide additional information about semen quality.	Conditional	Grade D
The information provided by SDF testing may guide the decision to use IUI or IVF/ICSI for future conception with cryopreserved sperm –in case both options are available–, and the choice of the optimal sperm freezing method.	Conditional	Grade D

SDF = sperm DNA fragmentation; RPL = recurrent pregnancy loss; ICSI = intracytoplasmic sperm injection; IUI = intrauterine insemination; IVF = in vitro fertilization.

§ Guideline development group (GDG) expert judgment; Strong recommendations imply that most individuals in that situation should receive the testing or intervention. Conditional recommendations imply that different choices might be appropriate for individual patients and that clinicians should help each patient reach a decision consistent with a patient-centered approach.

* Oxford Centre for Evidence-Based Medicine Levels of Evidence (OCEBM Levels of Evidence Working Group)

Grades of recommendations according to quality of evidence:

Grade A: consistent level 1 studies; Grade B: consistent level 2 or 3 studies or extrapolations from level 1 studies; Grade C: level 4 studies or extrapolations from level 2 or 3 studies; Grade D: level 5 or troubling inconsistent or inconclusive studies of any level.

Level 1 studies: systematic reviews with homogeneity of randomized controlled trials (RCTs) or level 1 diagnostic studies (1a); individual RCT with narrow confidence interval or validating cohort studies with good reference standards (2b).

Level 2 studies: systematic reviews with homogeneity of cohort studies or diagnostic studies (2a); individual cohort study or low quality RCT (2b), exploratory cohort study with good reference standards (2b).

Level 3: systematic reviews of case-control studies or moderate quality diagnostic studies (3a), individual case-control studies or non-consecutive diagnostic studies (3b).

Level 4: case-series or poor cohort/case-control studies or case-control diagnostic study.

Level 5: Expert opinion

http://www.cebm.net/oxford-centre-evidence-based-medicine-levels-evidence-march-2009/). Accessed June 7th, 2020.

Reprinted from: Esteves SC, Zini A, Coward RM, Evenson DP, Gosálvez J, Lewis SEM, Sharma R, Humaidan P. Sperm DNA fragmentation testing: Summary evidence and clinical practice recommendations. Andrologia. 2020 Oct 27:e13874. Epub ahead of print. This is an open access article distributed under the terms of the Creative Commons Attribution License. The license permits unrestricted use, distribution, reproduction in any medium, provided the original work is properly cited.

### Why and how to use the SFRAG guideline

The SGRAG guideline is unique as it unites reproductive urologists with vast clinical experience in diagnosing and treating male factor infertility. Moreover, for the first time, a group of scientists pivotal in developing the four major SDF assays used nowadays worked together. They deciphered each test’s technical aspects, making it easier to interpret the results and understand the intrinsic limitations of these assays. Furthermore, the SFRAG guideline includes an experienced reproductive endocrinologist with vast clinical experience, who added unique insights concerning the application of SDF testing in couples undergoing assisted reproduction.

**Figure 2:**
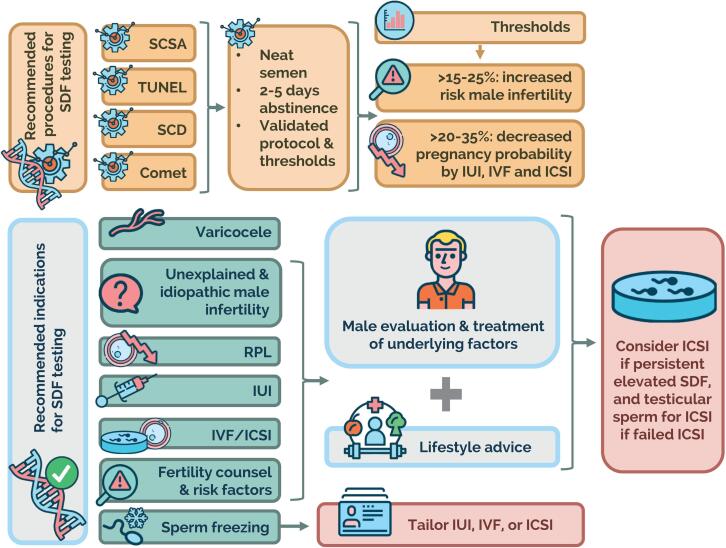
A Pictorial summary of the recommendations for sperm DNA fragmentation testing and possible management in couples with elevated sperm DNA fragmentation. IUI: intrauterine insemination; IVF: in vitro fertilization; ICSI: intracytoplasmic sperm injection; RPL: recurrent pregnancy loss. Reprinted from: Esteves SC, Zini A, Coward RM, Evenson DP, Gosálvez J, Lewis SEM, Sharma R, Humaidan P. Sperm DNA fragmentation testing: Summary evidence and clinical practice recommendations. Andrologia. 2020 Oct 27:e13874. Epub ahead of print. This is an open-access article distributed under the Creative Commons Attribution License. The license permits unrestricted use, distribution, reproduction in any medium, provided the original work is properly cited.

The guideline summarizes and critically appraises the most relevant studies published to date. Thus, for each recommendation, a strength rating based on both expert judgment and evidence levels is provided. The clinical scenarios warranting SDF testing are dissected, and the best evidence-based treatment practices are provided. Notably, the guideline emphasizes the central role of urologists in the evaluation of the infertile male partner and highlights the importance of corrective measures to improve the male reproductive health overall, and SDF in particular. Figure-2 summarizes the SFRAG guideline in a snapshot.

The primary goals of the SFRAG guideline are to provide clinicians -urologists, andrologists, gynecologists, and reproductive endocrinologists - with clear advice on best practices in SDF testing and treatment. Besides treating conditions known to impair fertility and SDF, like varicocele, the reproductive urologist may identify other factors associated with the SDF, including subclinical infections, systemic diseases, and unhealthy lifestyle factors. For couples who need assisted reproductive technology, the reduction in SDF rates may help improve success rates, and downgrade the complexity and cost of the method potentially, or even help achieve natural conception.

The SFRAG guideline statements were developed based on the best available evidence, with the grade of recommendation ranging from low to moderate. This thematic area still lacks high-quality studies, thus offering ample research opportunities. Such a guideline should be used as a tool to help standardize care, however, it does not mandate clinical care pathways. The SFRAG guideline is a clear, concise summary of best practices in SDF testing and treatment that represents an invaluable resource for a broad range of professionals providing infertility care.

### Data availability statement

This paper provides an abridged version of SFRAG guidelines, an open-access article distributed under the Creative Commons Attribution License. The license permits unrestricted use, distribution, reproduction in any medium, remixing, transformation, and building upon the material for any purpose provided the original work is properly cited. The full version can be found at https://onlinelibrary.wiley.com/doi/10.1111/and.13874.
